# Enhanced rhamnolipids production using a novel bioreactor system based on integrated foam-control and repeated fed-batch fermentation strategy

**DOI:** 10.1186/s13068-020-01716-w

**Published:** 2020-04-24

**Authors:** Ning Xu, Shixun Liu, Lijie Xu, Jie Zhou, Fengxue Xin, Wenming Zhang, Xiujuan Qian, Min Li, Weiliang Dong, Min Jiang

**Affiliations:** 1 State Key Laboratory of Materials-Oriented Chemical Engineering, College of Biotechnology and Pharmaceutical Engineering, Nanjing Tech University, Puzhu South Road 30#, Nanjing, 211816 People’s Republic of China; 2 Jiangsu Key Laboratory for Biomass-based Energy and Enzyme Technology, Huaiyin Normal University, Huai’an, People’s Republic of China; 3 Jiangsu National Synergetic Innovation Center for Advanced Materials (SICAM), Nanjing Tech University, Nanjing, People’s Republic of China

**Keywords:** Rhamnolipids, Ex situ foam control, Foam reduction, Repeated fed-batch

## Abstract

**Background:**

Rhamnolipids are the best known microbial-derived biosurfactants, which has attracted great interest as potential ‘‘green” alternative for synthetic surfactants. However, rhamnolipids are the major contributors to severe foam problems, which greatly inhibit the economics of industrial-scale production. In this study, a novel foam-control system was established for ex situ dealing with the massive overflowing foam. Based on the designed facility, foam reduction efficiency, rhamnolipids production by batch and repeated fed-batch fermentation were comprehensively investigated.

**Results:**

An ex situ foam-control system was developed to control the massive overflowing foam and improve rhamnolipids production. It was found that the size of individual bubble in the early stage was much larger than that of late fermentation stage. The foam liquefaction efficiency decreased from 54.37% at the beginning to only 9.23% at the end of the fermentation. This difference of bubble stability directly resulted in higher foam reduction efficiency of 67.46% in the early stage, whereas the small uniform bubbles can only be reduced by 57.53% at the later fermentation stage. Moreover, reduction of secondary foam is very important for foam controlling. Two improved designs of the device in this study obtained about 20% improvement of foam reduction efficiency, respectively. The batch fermentation result showed that the average volume of the overflowing foam was reduced from 58–640 to 19–216 mL/min during the fermentation process, presenting a notable reduction efficiency ranging from 51.92 to 73.47%. Meanwhile, rhamnolipids production of batch fermentation reached 45.63 g/L, and the yield 0.76 g/g was significantly better than ever reported. Further, a repeated fed-batch fermentation based on the overall optimization was carried out. Total rhamnolipids concentration reached 48.67 g/L with the yield around of 0.67–0.83 g/g, which presented an improvement of 62% and 49% compared with conventional batch fermentation by using various kinds of defoamers, respectively.

**Conclusions:**

The ex situ foam-control system presented a notable reduction efficiency, which helped greatly to easily solve the severe foaming problem without any defoamer addition. Moreover, rhamnolipids production and yield by repeated fed-batch fermentation obtained prominent improvement compared to conventional batch cultivation, which can further facilitate economical rhamnolipids production at large scales.

## Background

Biosurfactants produced from various low-carbon sources by microorganisms were increasingly viewed in last decades. As reported, the market for these ‘‘green” surfactants was $24 million in 2009 and was expected to reach to $2.8 billion by 2023 [1]. Rhamnolipids are a major class of biosurfactants that has the potential for commercial exploitation due to its excellent surface/interfacial properties. Rhamnolipids contain one or two l-rhamnose units linked to one or two β-hydroxy fatty acids, and it can accumulate at the interface to reduce the surface tension between two phases [2]. Due to their advantages over synthetic chemical surfactants (e.g., lower toxicity, better biodegradability, high specificity, constant effectiveness over wider range of pH and temperature), it possesses great potential in various fields ranging from cleaning agent in cosmetic industry, emulsifier or solubilizer in food-processing, oil-displacing agent for oil recovery or even cosolvent in the pesticide production [3].

Currently, rhamnolipids production on the large scale was still limited due to its low yields and high production costs [4]. Especially, the extreme foaming problem in aerobic fermentation was thought to be a main challenge [5, 6]. Severe foaming inhibits cell growth and product accumulation by reducing the working volume, bioavailability of substrates, mass transfer efficiency of oxygen and other adverse physical or biological effects [7, 8]. Moreover, massive foam overflowing from the gas outlet of bioreactor would have several detrimental impacts on the fermentation, such as loss of culture medium, rhamnolipids and cells, and high risk of contamination [8, 9].

In the past few years, extensive efforts have been made to avoid or control the foaming problem during rhamnolipids fermentation process [10]. The most common method is the addition of anti-foam agents [5, 11]. However, the extreme severe foam formation in rhamnolipids fermentation needs a massive amount of defoamer, which is costly and affects the recovery and quality of the rhamnolipids [12, 13]. Another extensively used method is mechanical breakers which are installed in the mechanical rotary devices mounted on top of the bioreactor [14]. However, it cannot effectively break rhamnolipids foam, and even may aggravate more serious problem by producing more stable secondary foam in the top space of bioreactor [13]. Moreover, anaerobic fermentation, solid-state fermentation and membrane-assisted bioreactor have also been applied to decrease the air flow to reduce the foam formation during the fermentation [15–17]. In these cases, reduction of the aeration could control severe foaming, but it impaired the rhamnolipids productivity because of oxygen limitation [17]. In addition to the above strategies, the synchronous separation or removal of rhamnolipids during the fermentation was also investigated [18, 19]. Even though these techniques could effectively control the foam formation, there were still many problems to be solved. The most important was the lower yield and high cost of complicated setup.

Rhamnolipids were mainly produced in stationary phase and usually need a few days for one production cycle in batch fermentation [20]. Thus, extension of production cycle and maintenance of high productivity at late stage are important to improve the rhamnolipids production efficiency. Considering these, fed-batch fermentation was considered as more efficient in maintaining the biomass growth rate and rhamnolipids productivity [19, 21–23]. However, rhamnolipids are both the target product and major contributor to severe foam problems, and more rhamnolipids production would cause more severe foam problem, needing more efficient foam-controlling strategy [6]. Generally, when the concentration of rhamnolipids reached about 500 mg/L, the foam fully filled the whole reactor and began to overflow from the exhaust-gas line along with the airflow [12]. Thus, an integrated strategy of maximizing production efficiency by fed-batch fermentation while achieving efficient foam-control is crucial for the large-scale rhamnolipids production.

In this study, an integrated foam-control system was designed to deal with the serious foaming problem. Based on the designed facility, the characterization of in situ severe foam and the foam reduction efficiency was first evaluated. The rhamnolipid production efficiency was also investigated compared with traditional foam-controlling method in batch fermentation. At last, the combined strategy for suitable foam-control and repeated fed-batch rhamnolipids fermentation were conducted and their respective performances were compared.

## Results and discussion

### Construction of foam-control system for improving rhamnolipids batch fermentation production

**Fig. 1:**
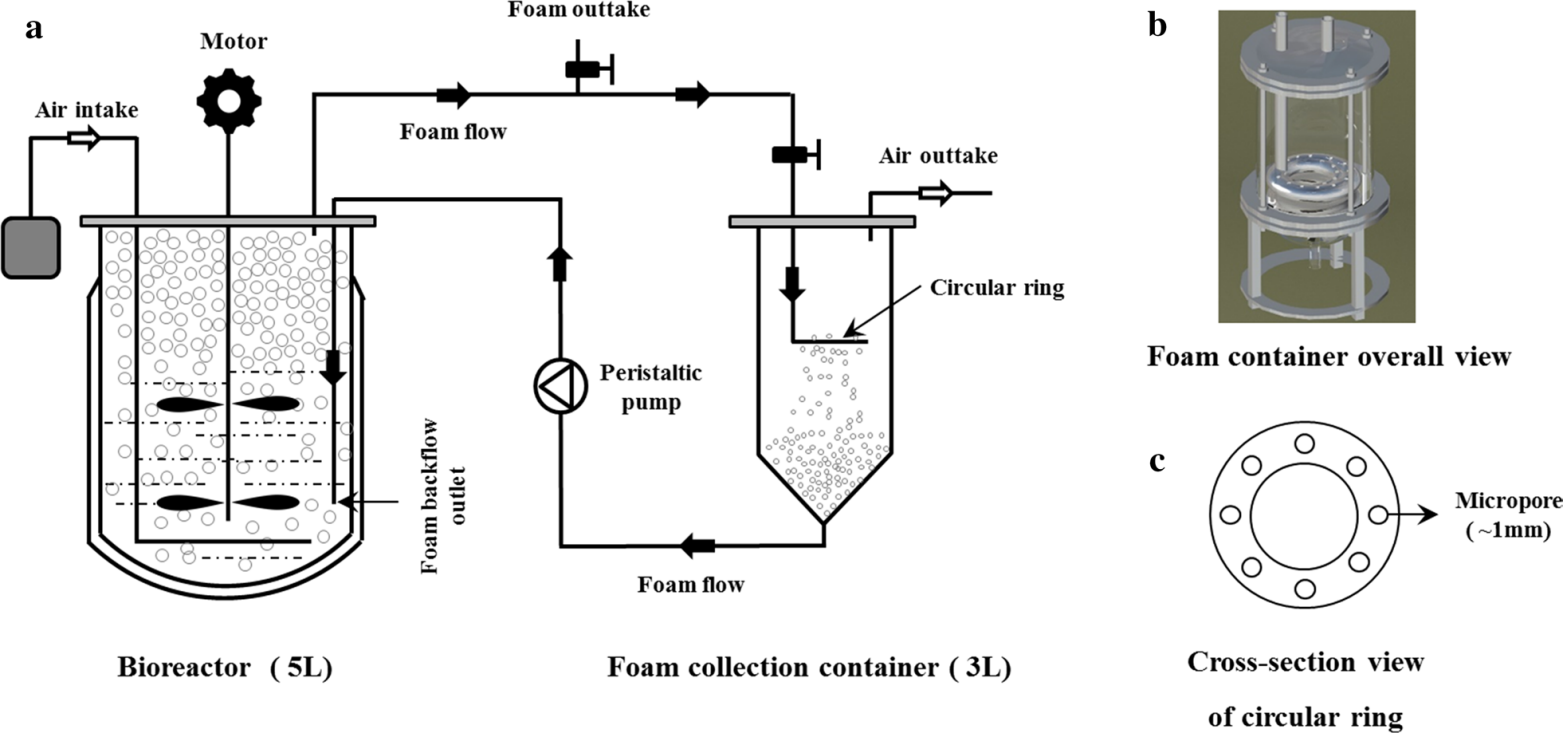
Diagram of integrated system for foam-control and repeated fed-batch fermentation (**a**). During the fermentation, foam formed in the head space of bioreactor and streamed into collecting container (**b**) under the driving force of air flow. The circular ring (**c**) with some ⌀1 mm micropores could break the foam into liquid. After breaking, little residual foam and broth were pumped back into the bioreactor by peristaltic pump

As stated, an ex situ foam-control system was developed to collect the in situ foam accumulation data and to control the massive overflowing foam in rhamnolipids fermentation (Fig. 1). During the fermentation, overflowing foam which formed in the head space of bioreactor streamed into the collecting container under the driving force of air flow. The circular ring (Fig. 1c) with some ⌀1-mm micropores was the key appliance which could efficiently break the foam into liquid. After breaking, little residual foam and broth were pumped back into the bioreactor.

**Fig. 2:**
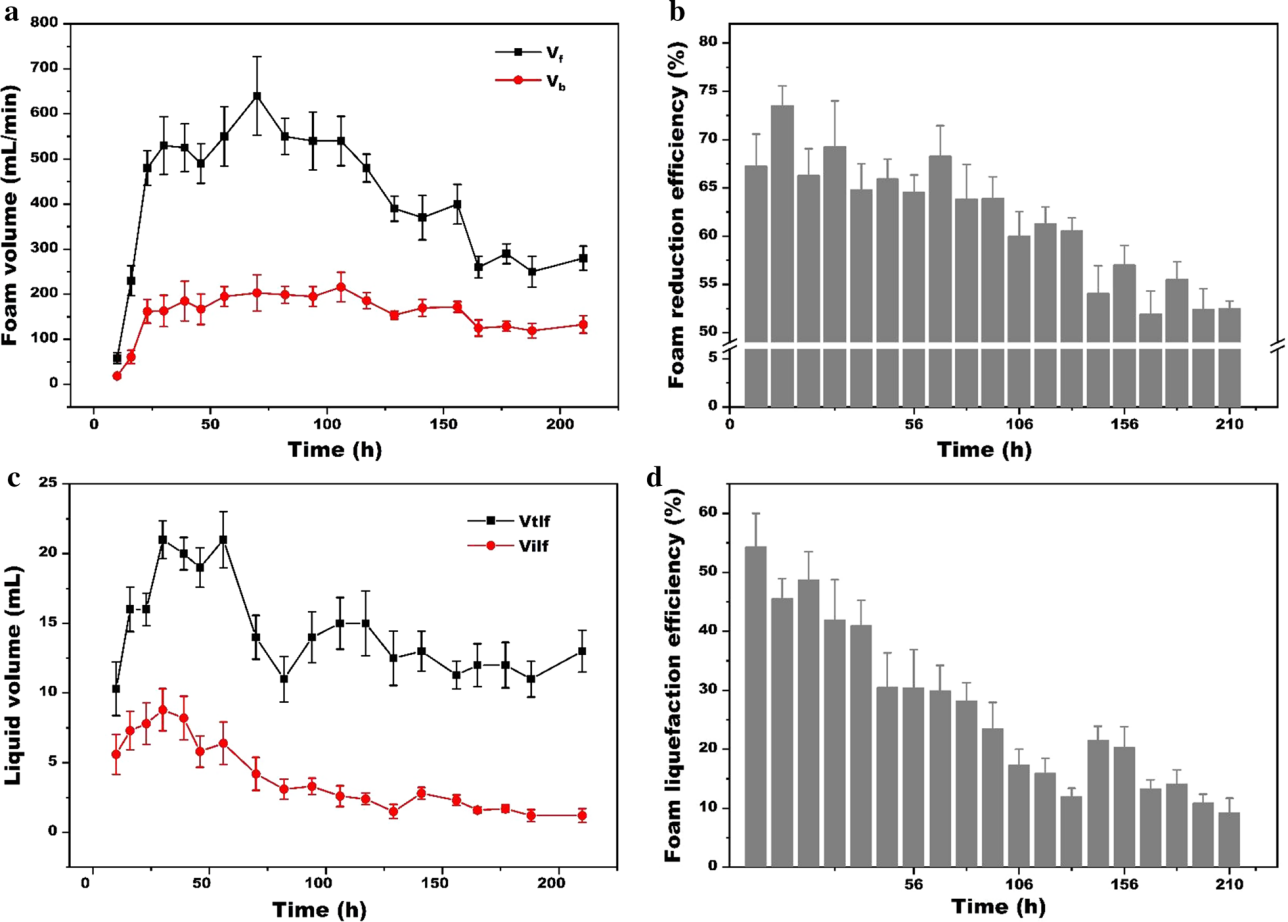
Foam reduction efficiency of foam-control system during rhamnolipids batch fermentation. *V*_f_, the volume of the overflowing foam which streamed through the outtake in 1 min; *V*_b_, the volume of residual foam streamed through micropore of circular ring in 1 min; *V*_tlf_, left the foam sample stilly and recorded the liquid volume when all foam liquefies into liquid; *V*_ilf_, when foam passed through the foam breaker, most bubbles would collapse into liquid and record this liquid volume immediately

Starting from 10 h, the volume of overflowing foam at different times of fermentation is shown in Fig. 2a, b. During the whole fermentation, micro-pore breaker presented a notable reduction efficiency ranging from 51.92 to 73.47%, which reduced the volume of overflowing foam ranging from 58–640 to 19–216 mL/min. The high reduction efficiency notably reduces the collection container volume to only 20% of the bioreactor, which was much smaller than reported installation [10]. Moreover, the whole ex situ foam-control system was simple and easy to operate, which helped to greatly reduce the production costs.

In order to investigate the effect of foam-control system on rhamnolipids production, two 5-L bioreactors were run simultaneously with 2.5-L working volume and 60 g/L colza oil. One bioreactor was used as the control, which used chemical defoamers to prevent the severe foam from overflowing the fermentation tank. As shown in Table 1, the rhamnolipids concentration of foam-control system reached 45.63 g/L, an improvement of 52.30% compared to regular method. Moreover, the rhamnolipids yield reached 0.76 g/g, which presented a significant improvement over the previous reports [14, 18, 19, 24]. This improvement might be benefited from the elimination of toxic defoamer [11], sufficient supply of dissolved oxygen (DO) [24], which can sustain good cell growth and rhamnolipids biosynthesis. Moreover, by adjusting the rotation speed of the peristaltic pump (precise speed data not shown), residual foam and liquid were timely pumped back to the bottom of the fermentation broth with nearly no negative effects on cell growth and metabolism. In conclusion, the result showed that this foam-control system can fully meet the requirements for rhamnolipids batch fermentation.

**Table 1:** Performance and comparisons of rhamnolipids production using anti-foam agents and foam-control system

Comparisons	Anti-foam agents	Foam-control system
Interval of defoaming agent (h)	2.0–6.0	No need
Dosage (μL)	20–100	–
Total amount of defoamer (mL)	4.5 ± 0.5 mL	–
Maximum dry biomass (g/L)	7.61 ± 1.13	9.24 ± 0.92
Rhamnolipid (g/L)	29.96 ± 0.89	45.63 ± 3.36
Yield (g/g)^a^	0.50 ± 0.07	0.76 ± 0.11

^a^ Yield of rhamnolipids production was calculated based on total colza oil consumption

### Foam in situ accumulating characterization and foam reduction efficiency

At the beginning of batch cultivation, there were only a small amount of bubbles floated on the surface of culture medium. These bubbles spontaneously collapsed and could not accumulate until about 6 h after inoculation. From then on, the bubbles slowly accumulated and formed stabilized foam, which consequently resulted in the increase of volume and height of the foam in the bioreactor. At about 10 h, the foam fully filled the whole reactor and began to overflow from the exhaust-gas line along with the airflow. At this moment, the concentration of rhamnolipids was about 610 mg/L, which was consistent with previous results [12].

As shown in Fig. 2a, b, foam reduction was not a stable value during the whole fermentation process. According to the volume of overflowing foam, we divided the process into two periods (Table 2). During 10 to 24 h, the foam volume sharply increased from 58 to 480 mL/min with a higher growth rate of 32.19 mL/h. When these large bubbles passed through the micropore of the circular ring (foam breaker, Fig. 1c), the foam volume would be reduced from 58, 230, 480 mL/min to only about 20, 60, 160 mL/min, leading to 67.24, 73.47%, 66.25% reduction efficiency at 10, 16 and 24 h, respectively. In the next 48 h, the foam volume increased slowly and the average reduction efficiency in this period is 66.55%, which is very close to the first 24 h. At about 70 h, the volume of overflowing foam reached the maximum speed of 640 mL/min. At this time, the foam breaker could reduce the foam volume to about 200 mL/min with a reduction efficiency of 68.28%. After that, the volume of overflowing foam slowly decreased to a stable value of 260 mL/min, and the reduction efficiency also gradually decreased to about 51.92% at 165 h.

**Table 2:** Comparisons of foam reduction efficiency in different fermentation stages

Reduction efficiency (%)	Fermentation time (h)	
	0–70	70–210
Maximum	73.47 ± 2.06	67.46 ± 2.92
Minimum	64.54 ± 1.80	51.92 ± 2.40
Average	63.88 ± 2.27	57.53 ± 4.56

**Fig. 3:**
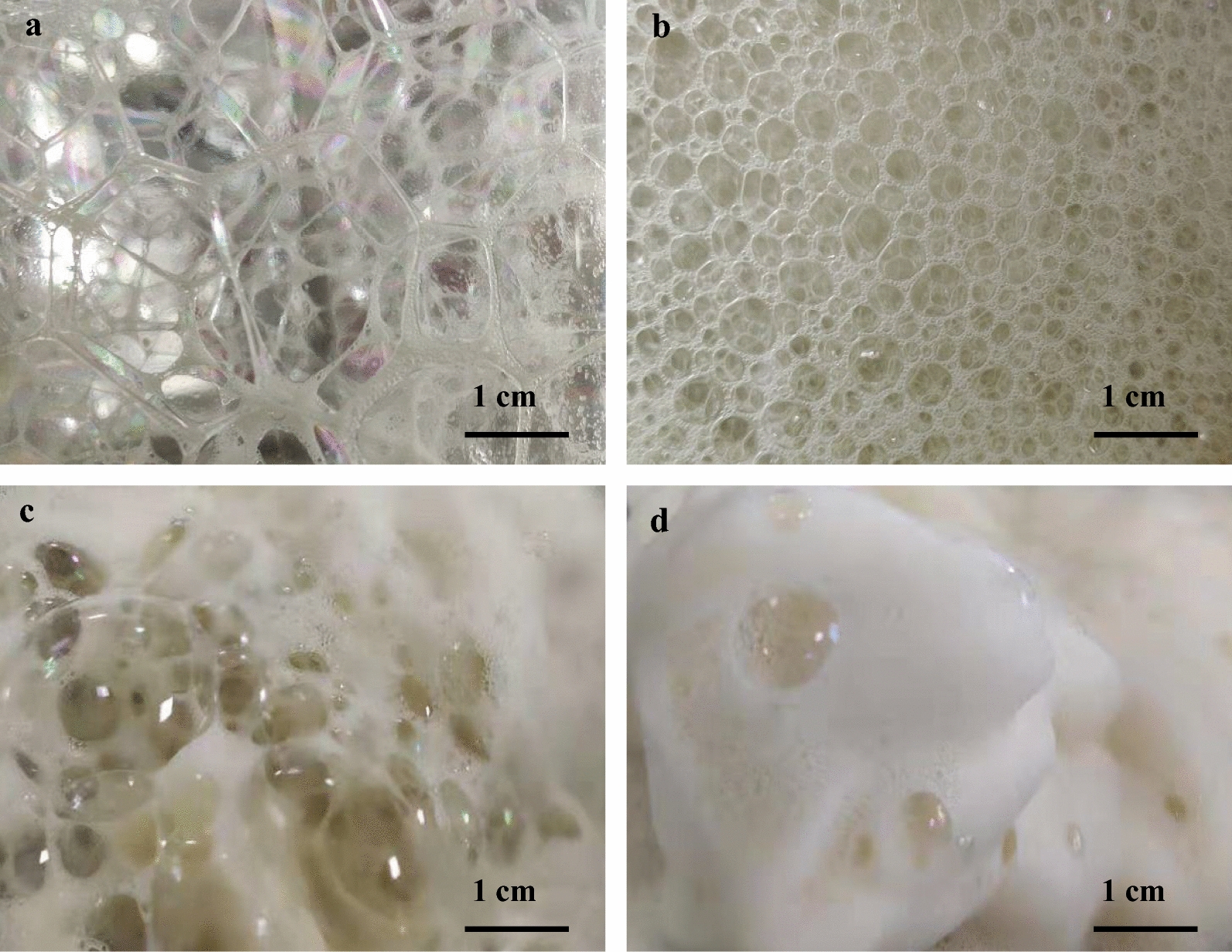
Foam morphology before and after breaking in different stages of the fermentation. Foam morphology of early stage, before (**a**) and after breaking (**c**), and foam morphology of later stage, before (**b**) and after breaking (**d**)

According to previous research, when foam flowed quickly with the air flow, the mechanical force produced by the sharp gap of tiny micropores [14, 25]. We found that individual bubbles with different size exhibit different reduction efficiency. As seen in Fig. 3a, large bubbles are incompact, irregular and rich in water phase in the early stage (10–70 h). During 70–210 h, the size of the individual bubble gradually decreased and these small uniform bubbles (Fig. 3b) became more denser than those of the previous stage. When the overflowing foam passed through the micropore of the circular ring (foam breaker), the lager bubbles of early stage collapsed more efficiently (Fig. 3c) with a higher average reduction of 67.46% (Table 2), whereas the small uniform bubbles can only be reduced by 57.53% at the later fermentation stage (Fig. 3d).

To confirm this, the foam liquefaction efficiency (FLE) was assayed. As expected, the FLE decreased from 54.37% at the beginning, to only 9.23% at the end of the fermentation (Fig. 2c, d). It means that large bubbles in early stage were easier to collapse and liquify than that of later stage which directly resulted in higher foam reduction efficiency in the early fermentation stage. This result is consistent with previous study, which used perforated plate as defoamers to break the flowing foam [26]. In the case that the rapid bubble expansion occurs at the top surface of the foam, surface excess will decrease, surface tension will increase and the film thickness of the bubble will decrease. The thinner film of large-diameter bubbles easily reached its critical value. Therefore, these large bubbles have weak stability and burst easily.

### Inhibition of secondary foam formation to improve foam reduction efficiency

When bubbles on a liquid–gas or solid–gas interface rupture, the general expectation is that the bubbles vanish and retract rapidly until they become part of the interface [25]. However, for a large range of fluid parameters, interfacial bubbles can create numerous tiny bubbles rather than vanish when they rupture [27]. As shown in Fig. 3, both large and small bubbles at different fermentation stages produced numerous tiny bubbles after breaking, and only part of the foam liquefied into broth (Fig. 2c). These tiny bubbles were secondary bubble which was extremely stable and significantly increased the difficulty of foam controlling [24, 28]. Therefore, controlling and reducing of the secondary foam will help greatly in improving foam reduction efficiency during the fermentation.

**Fig. 4:**
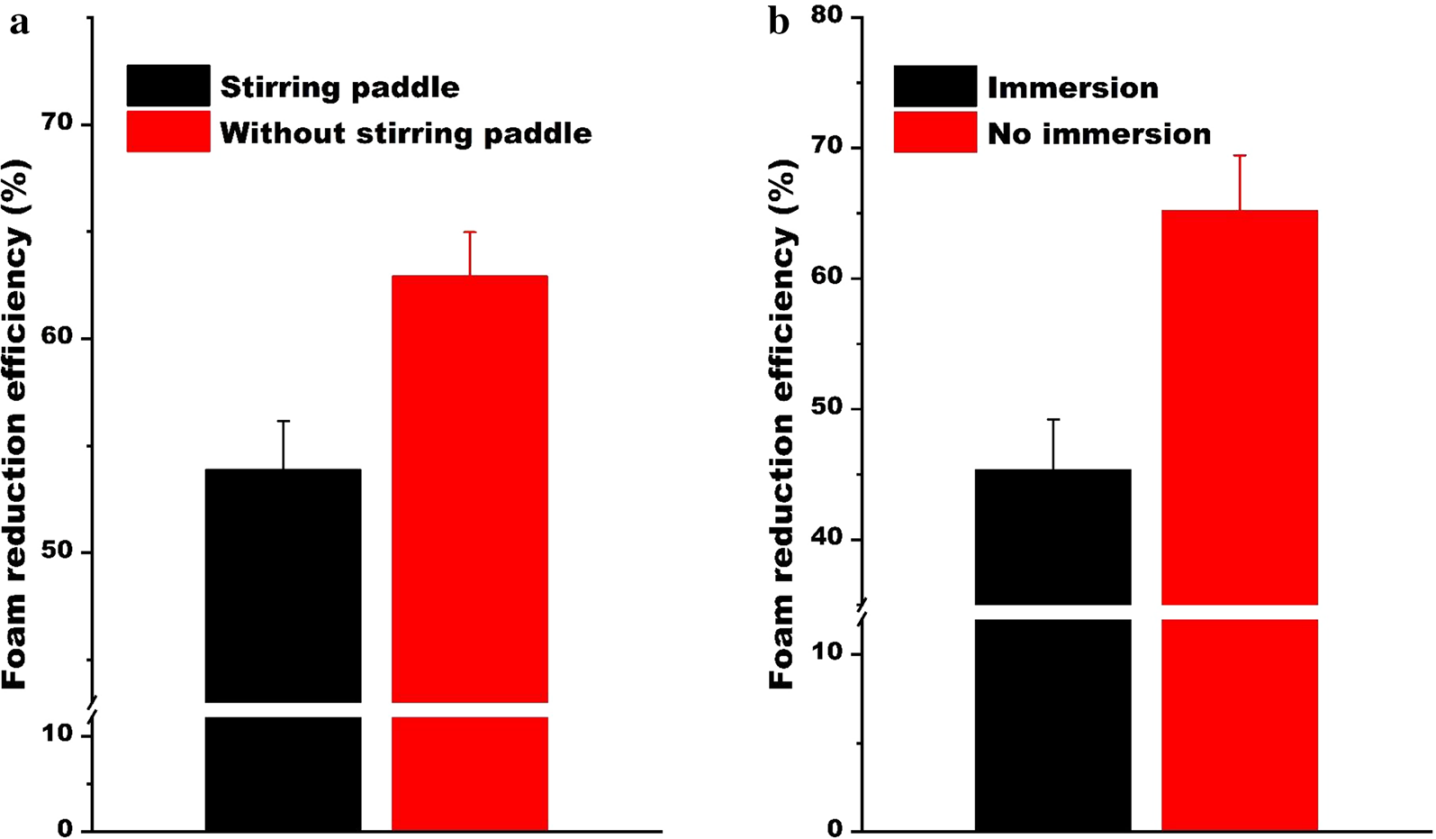
Inhibition of secondary foam formation to improve foam reduction efficiency

In this study, two measures were taken to reduce the generation of secondary bubble. First, the stirring paddle which was usually set at the top of the fermentation tank was removed (Fig. 1a). By removing the stirring paddle, the foam reduction efficiency gained about 10% enhancement which helps greatly in foam control (Fig. 4a). Another measure for reducing the secondary foam was to raise the height of the circular ring to prevent it from immersing in residual foam, which would avoid the negative effect of previous residual foam on subsequent foam breaking. The result showed that it improved the reduction efficiency from 45.35 to 65.23% (Fig. 4b). The first reason for the enhancement of foam-breaking performance is that the film retracts speed in the air is faster than in the residual foam when the bubbles ruptured [27]. Moreover, the foam in the pipe was discontinuous and separated by air. When the air was released from the micropore, it would produce some bubbles in the previous remaining foam, especially the large bubbles (Fig. 3c, d). However, similar situation did not occur when the ring was suspended in the air and was not immersed in foam.

### Repeated fed-batch fermentation for rhamnolipids production

**Fig. 5:**
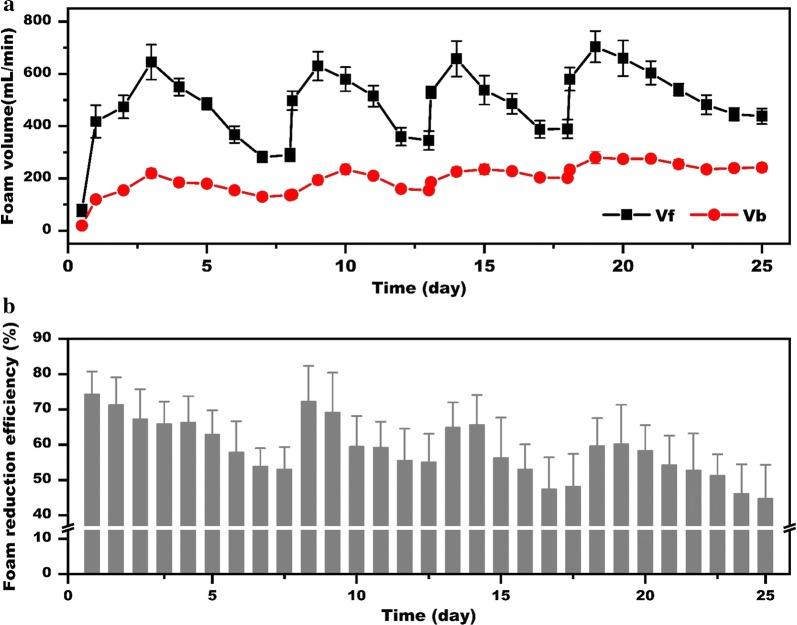
Foam reduction efficiency of foam-control system during the repeated fed-batch fermentation

The applicability of this foam-control system in repeated fed-batch fermentation was further investigated. Once the fresh fermentation medium was added into the bioreactor, the small bubbles of the end initial fermentation (Fig. 3b) changed into large bubbles which was similar to the early stage of batch fermentation (Fig. 3a). And the volume of overflowing foam increased rapidly in the next 48 h, just like the early stage of the initial fermentation progress. Interestingly, the maximum volume of the overflowing foam in the first and second feed batch cycle was very close to that of initial fermentation (Fig. 5a). Due to the higher reduction efficiency (Fig. 5b), the severe foam problem could be fully solved by the system in first and second fed batch cycles. However, the foam problem became more and more difficult to be controlled in the next cycles. As the fermentation continued, the finished foam volume of individual cycle greatly increased to 438 ± 30 mL (Fig. 5a), which was much higher than before. Moreover, the reduction efficiency of the third cycle decreased to only 44.74% (Fig. 5b). At the end of the third cycle, there was much more secondary foam in the collection container which needs to constantly increase the pump speed to ensure the timely foam reflux. Thus, the foam reduction efficiency would gradually decrease along with the feeding times, and too long continuous fermentation would make it very difficult to control the foam.

**Fig. 6:**
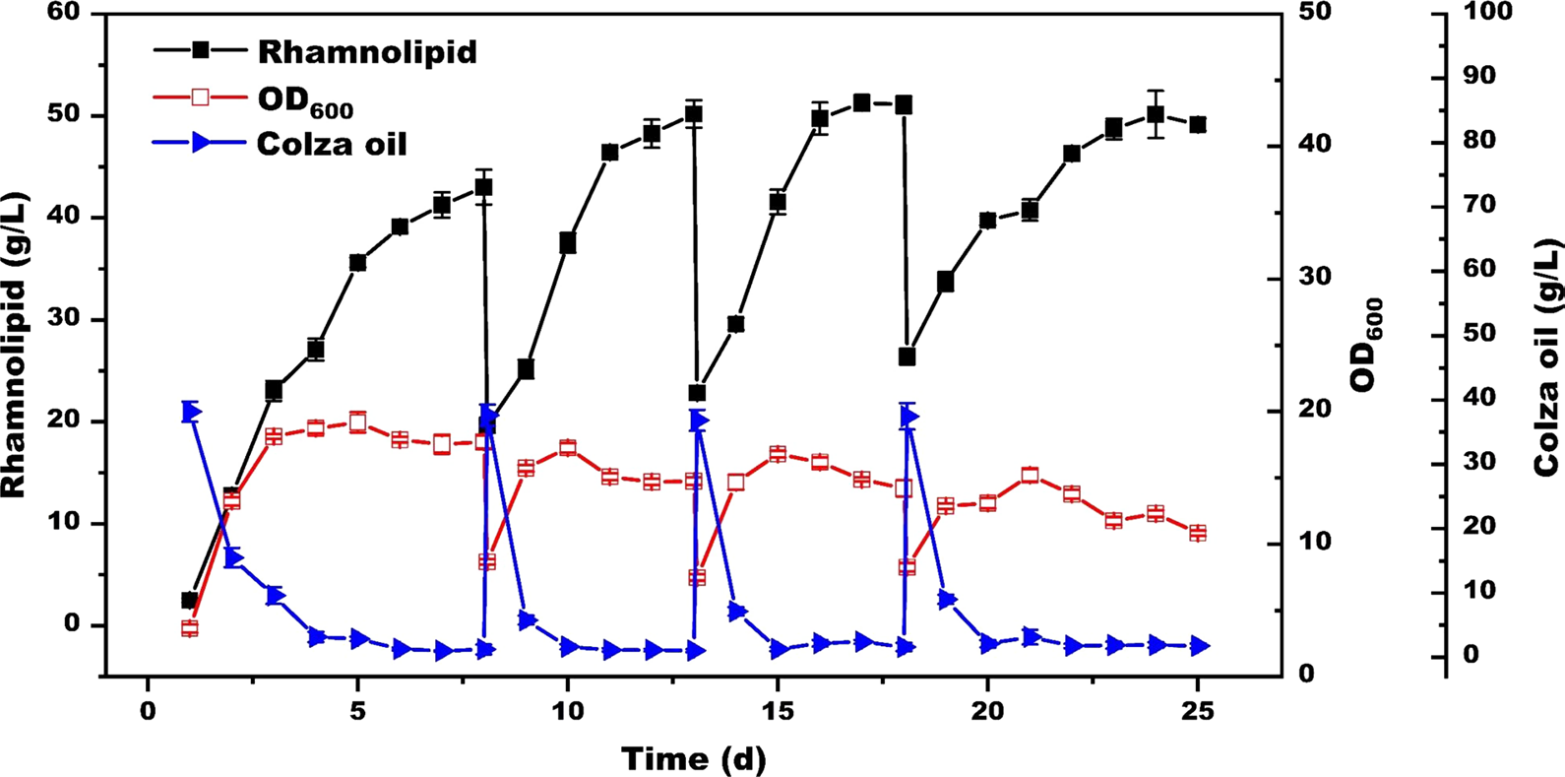
Rhamnolipids production with repeated fed-batch fermentation strategy in foam-control system

According to the foam-control situation, three cycles were finally adopted for rhamnolipids repeated fed-batch fermentation. As shown in Fig. 6, our strategy would sustain good productivity and high rhamnolipids production for 25 days. During the repeated fed-batch fermentation, substrate consumption rate was faster and microorganisms were restored well with higher growth rate than the initial fermentation. Rhamnolipids concentration finally reached about 50 g/L at the end of every cycle of repeated fed-batch fermentation, which was even higher than batch fermentation. Moreover, the yield was well-maintained in a range of 0.67–0.83 g/g substrate compared with batch cultivation, respectively. In addition to elimination of toxic defoamer addition, sufficient supply of dissolved oxygen [27], feeding of fresh medium as well as dilution of the culture broth also contributed greatly in maintaining cell growth and high rhamnolipids productivity over time [5, 22, 23]. Finally, the overall rhamnolipids reached 48.67 g/L with an average yield of 0.75 g/g substrate within one repeated fed-batch fermentation period, leading to an increase of 62% and 49% over the conventional batch cultivation, respectively. This well-maintained productivity and the high yield were remarkably better than a similar foam-control system [24]. More important, this combined strategy was much more economical and eco-friendly compared with the traditional method [5, 11, 12], and gave more feasible option for industrial application.

## Conclusion

In this study, an ex situ foam-control system was developed to solve the severe foam problem during rhamnolipids fermentation. Overall improvement of the installation realized a notable reduction efficiency ranging from 51.92 to 73.47%, which provided a simple and economical strategy to control the severe foam problem. After repeated fed-batch fermentation for 25 days, the overall rhamnolipids reached 48.67 g/L with an average yield of 0.75 g/g, leading to the improvement of 62% and 49% over the conventional batch cultivation using various kinds of defoamers, respectively. Therefore, this well-reproducible foam-control system can efficiently facilitate economical production of rhamnolipids at large scales.

## Methods

### Strain and culture medium

*Pseudomonas aeruginosa* KT1115, which was isolated from oil-contaminated soil samples, was employed in this work. Luria–Bertani (LB) medium containing 10 g/L tryptone, 5 g/L yeast extract and 10 g/L NaCl was used for seed culture. The fermentation medium contains: 60 g/L colza oil, 6 g/L NaNO_3_, 3 g/L yeast extract, 1 g/L KH_2_PO_4_, 1 g/L Na_2_HPO_4_, 0.1 g/L CaCl_2_ and 0.1 g/L MgSO_4_. The pH of the fermentation medium was adjusted to 7.0 using 1 M NaOH solution prior to autoclaving. All chemicals and solvents were biochemical or analytical grade and purchased from Sangon Biotech (Shanghai) Co., Ltd.

### Design and construction of foam-control system

An ex situ foam-control system was designed to control the massive overflowing foam in rhamnolipids fermentation. As shown in Fig. 1, an exhaust-gas line on the top of the bioreactor was connected to a foam collecting container composed of a simple 3-L glass bottle equipped with a circular ring for breaking the foam and a bottom outlet for foam–liquid broth recirculation. The circular ring (Fig. 1c) was installed in the middle of the vertical tank and there are some ⌀1-mm micropores in the circular ring, which could efficiently break the foam. Initial foam formed in the bioreactor head space streamed into collecting container under the driving force of air flow. When the foam went through the micropore, the loosened larger diameter bubbles of the foam would collapse or be broken into dense smaller bubbles and enriched liquid. After breaking, the collapsed foam liquid together with small amount of residual foam was pumped back to the bottom of the bioreactor using a peristaltic pump (BT300-2J, Longer, China). The off-gas released from the foam bubbles was separately led off through the air outtake on the top of the collecting container.

### Rhamnolipids fermentation

For batch fermentation, single *P. aeruginosa* KT1115 colony on LB agar plate was inoculated into 150 mL LB medium in a 500-mL Erlenmeyer flask and cultured in a shaking incubator (MQD-B1R, Minquan Instrument Co., Ltd, Shanghai) at 30 °C with 200 rpm for 36 h. This culture broth was used as seed medium and inoculated into 2.5 L fermentation medium in a 5 L bioreactor (T&J Bio-engineering Co., Ltd, shanghai) equipped with an integrated process control system for temperature, pH, DO, and airflow. The fermentation was performed at 30 °C for 7 days. The initial air flow rate was set at 1 vvm and the initial agitation rate was set at 400 rpm. During the fermentation process, air flow and agitation rate should be modified on account of the amount of the foam formation.

For the semi-continuous fermentation, when the concentration of rhamnolipids reached the stable phase at about 7 days, 1.5 L fermentation broth was took out from bioreactor in the form of foam through the foam outtake (Fig. 1). The 1.0-L residual fermentation broth was used as the seed in the next cycle of fermentation. Then, 1.5 L fresh sterilized fermentation medium was added into the bioreactor and cultured at 30 °C for 4 days. After that, the next fermentation cycle was carried out in the same way as mentioned above until five cycles completed. During semi-continuous fermentation, rate of air flow and agitation should also be adjusted appropriately on account of the changes of the total foam.

### Biomass and rhamnolipids measurement

During fermentation, samples were collected for the analysis of cell and rhamnolipid concentrations every 24 h. The sample of fermentation broth (1 mL) was mixed with *n*-hexane in 1:1(v/v) ratio and vigorously shaken for 5 min to extract the residual colza oil. The mixture was then centrifuged at 12,000 rpm for 10 min and the broth was separated into three parts: (a) top layer of oil-rich *n*-hexane supernatant solution, (b) middle layer of cell-free culture broth (labeled as ‘‘Cell-free’’) and (c) the sediment (cell components). Then top layer of the fat-rich *n*-hexane solution was removed.

The optical density was determined by resuspending cell biomass in physiological saline to measure absorption at 600 nm using a Spectrophotometer UV2800 (Hengping, China). The optical density was read against the physiological saline as a blank. For dry cell weight measurement, the (c) the sediment was washed with 1 mL of distilled water three times. After centrifugation, the sediment was dried at 70 °C until constant weight was reached. For rhamnolipids measurement, the rhamnolipid concentrations were measured by the anthrone method at 620 nm [9, 11]. A correction factor of 2.21 was applied to compensate the extra mass of the lipidic portion of rhamnolipids. The correction factor was calculated based on the HPLC–MS analysis which gave the proportion of every rhamnolipid congeners in the sample. Both measurements were done in triplicate.

### Evaluation of in situ foam formation ability

During fermentation, foam samples were collected for the evaluation of in situ foam formation ability. The foam sample can be taken from foam outtake by blocking the foam flow to collecting container (Fig. 1). Then, measured the volume of the foam in 1 min with a measuring cylinder and recorded the total volume of foam (*V*_f_). All the foam samples were collected at the air flow rate of one volume of gas per volume of liquid per minute (VVM) and 300 rpm agitation.

### Evaluation of foam reduction efficiency

Using silicone tube to connect another circular ring to the foam outtake to evaluated the foam-breaking efficiency. When the overflowing foam streamed through micropore of circular ring, the foam would be collapsed and the residual foam volume (*V*_b_) was recorded. The foam reduction efficiency (FRE) was calculated according to Eq. (1):

1 $${\text{FRE}}\left[ {\text{\% }} \right] = \frac{{V_{\text{f}} - V_{\text{b}} }}{{V_{\text{f}} }} \times 100\% .$$

### Evaluation of foam liquefaction efficiency

Stopped the foam flow to the collecting container, then the foam would stream through the outtake. The foam was collected in 1 min with a measuring cylinder and the foam sample was left still until all foam liquefies into liquid. The volume of the liquid broth was recorded as *V*_tlf_. Using silicone tube to connect the circular ring to the foam outtake and the foam was collected in 1 min with a measuring cylinder. When the overflowing foam streamed through micropore of circular ring, some bubbles would liquify into liquid broth. The volume of the liquid (*V*_ilf_) was recorded immediately and the foam liquefaction efficiency was calculated according to Eq. (2):

2 $${\text{FLE}}\left[ {\text{\% }} \right] = \frac{{V_{\text{ilf}} }}{{V_{\text{tlf}} }} \times 100\% .$$

## Data Availability

The information about accession numbers is given in the manuscript.

## Abbreviations

FRE: Foam reduction efficiency
FLE: Foam liquefaction efficiency
